# Altered 2-thiouridylation impairs mitochondrial translation in reversible infantile respiratory chain deficiency

**DOI:** 10.1093/hmg/ddt309

**Published:** 2013-06-28

**Authors:** Veronika Boczonadi, Paul M. Smith, Angela Pyle, Aurora Gomez-Duran, Ulrike Schara, Mar Tulinius, Patrick F. Chinnery, Rita Horvath

**Affiliations:** 1 Institute of Genetic Medicine, Newcastle University, Central Parkway, Newcastle upon TyneNE1 3BZ, UK; 2 Department of Paediatric Neurology, University of Essen, Hufelandstraße 55, Essen45122, Germany; 3 Department of Paediatrics, The Sahlgrenska Academy, University of Gothenburg, Box 400, GöteborgSE-405 30, Sweden

## Abstract

Childhood-onset mitochondrial encephalomyopathies are severe, relentlessly progressive conditions. However, reversible infantile respiratory chain deficiency (RIRCD), due to a homoplasmic mt-tRNA^Glu^ mutation, and reversible infantile hepatopathy, due to tRNA 5-methylaminomethyl-2-thiouridylate methyltransferase (TRMU) deficiency, stand out by showing spontaneous recovery, and provide the key to treatments of potential broader relevance. Modification of mt-tRNA^Glu^ is a possible functional link between these two conditions, since TRMU is responsible for 2-thiouridylation of mt-tRNA^Glu^, mt-tRNA^Lys^ and mt-tRNA^Gln^. Here we show that down-regulation of TRMU in RIRCD impairs 2-thiouridylation and exacerbates the effect of the mt-tRNA^Glu^ mutation by triggering a mitochondrial translation defect *in vitro*. Skeletal muscle of RIRCD patients in the symptomatic phase showed significantly reduced 2-thiouridylation. *S*upplementation with l-cysteine, which is required for optimal TRMU function, rescued respiratory chain enzyme activities in human cell lines of patients with RIRCD as well as deficient TRMU. Our results show that l-cysteine supplementation is a potential treatment for RIRCD and for TRMU deficiency, and is likely to have broader application for the growing group of intra-mitochondrial translation disorders.

## INTRODUCTION

Mitochondrial diseases are a large and clinically heterogeneous group of disorders that result from deficiencies in cellular energy production and affect at least 1 in 5000 of the population. The underlying genetic defect in many patients remains unknown and there are no effective treatments (1,2). Most mitochondrial diseases are progressive conditions and lead to premature death. However, there is a unique condition, reversible infantile cytochrome *c* oxidase (COX) deficiency [or reversible infantile respiratory chain (RC) deficiency, RIRCD; OMIM# 500009], caused by the homoplasmic m.14674T>C/G mutation in the mt-tRNA^Glu^ gene, showing spontaneous recovery during early childhood (3–5). Affected children uniformly present with severe muscle weakness, often requiring assisted ventilation in the first days or weeks of life. If they survive the first months of life, they improve spontaneously, and recover fully by 2 or 3 years of age. The m.14674T>C/G mutation is thought to impair mitochondrial translation, as reflected by ragged red fibres/COX-negative fibres and multiple RC defects in skeletal muscle. The steady-state level of mt-tRNA^Glu^ was low in early biopsies (16–30%), but a slight increase occurred in the follow-up muscle biopsies, when the children were almost asymptomatic and remained low (30–60%) in primary fibroblasts (3). The slight recovery of the steady-state level of mt-tRNA^Glu^ in the face of dramatic clinical improvement indicates that, either this mild increase is sufficient to regain normal mitochondrial translation or other mechanisms downstream of mt-tRNA^Glu^ are responsible for the clinical and biochemical recovery. Low levels of mt-tRNA^Glu^ in muscle from clinically healthy mothers strongly suggest that the down-stream effects are able to ameliorate both the biochemical and clinical phenotype.

Although previous data provide strong evidence for a pathogenic role of m.14674T>C/G, they do not explain why all patients develop severe isolated myopathy in the neonatal period and, most importantly, what triggers the timed spontaneous recovery. Another unanswered question is why clinical symptoms manifest only in ∼30% of individuals carrying the homoplasmic m.14674T>C/G (3). However, no clear-cut nuclear modifiers of mtDNA disease have been identified to date (6).

RIRCD is not the only reversible mitochondrial disease. Autosomal-recessive mutations in a tRNA 5-methylaminomethyl-2-thiouridylate methyltransferase (*TRMU*, OMIM*610230, also known as *MTU1, MTO2)*, which is responsible for the 2-thiouridylation of mt-tRNA^Glu^, mt-tRNA^Gln^ and mt-tRNA^Lys^, but not of any other mt-tRNAs cause a severe but reversible infantile hepatopathy (7,8). Infants with reversible hepatopathy develop symptoms between 2 and 4 months of age, but if they survive this phase of liver failure, they recover and develop normally (8). The disease course and age of manifestation in TRMU deficiency shows remarkable similarities to RIRCD (5).

Recently, autosomal-recessive mutations were reported in infantile partially reversible hypertrophic cardiomyopathy in the gene *MTO1* (OMIM*614667) encoding the enzyme that catalyzes the 5-carboxymethylamino-methylation (mnm5s2U34) of the same nucleotide (U34) of the wobble position that is affected in TRMU deficiency for mt-tRNA^Glu^, mt-tRNA^Gln^ and mt-tRNA^Lys^ (9). Mutations in the glutamyl-tRNA synthetase (*EARS2*, OMIM*612799) cause early onset severe neurological disease (leukoencephalopathy involving the thalamus and brainstem with high lactate, LTBL) and 8 out of 12 patients showed clinical improvement and stabilization after 1 year of age (10).

The age-dependent, partially reversible clinical presentation and the impairment of mt-tRNA^Glu^ strongly suggest a possible pathophysiological link underpinning the spontaneous improvement in these mitochondrial conditions. We hypothesize that an impaired 2-thiouridylation in infants contributes to the clinical manifestation of RIRCD, therefore decided to study whether down-regulation of TRMU recapitulates the biochemical defect in RIRCD. Defining the common mechanism would not only suggest new avenues for treatment in these reversible disorders, but could also have more general relevance for the growing group of intra-mitochondrial translation defects.

## RESULTS

### 2-Thiouridylation pattern in RIRCD patient cells

To investigate whether the homoplasmic m.14674T>C/G mt-tRNA^Glu^ mutation impairs 2-thiouridylation of mt-tRNA^Glu^ in fibroblasts and myoblasts of a patient with RIRCD myopathy, we performed high-resolution northern blots by incorporating N-acryloylamino phenyl mercuric chloride (APM) into the gels, which enabled us to separate thiolated and non-thiolated tRNA species (11). We used probes for the three mt-tRNAs (Glu, Lys, Gln) undergoing 2-thiouridylation by TRMU, and also probed for cytoplasmic tRNA^Lys^, and 5S rRNA as non-thiolated controls in RIRCD cells, TRMU-deficient patient cells and normal controls. We studied both steady-state levels and level of thiolation.

**Figure 1.**
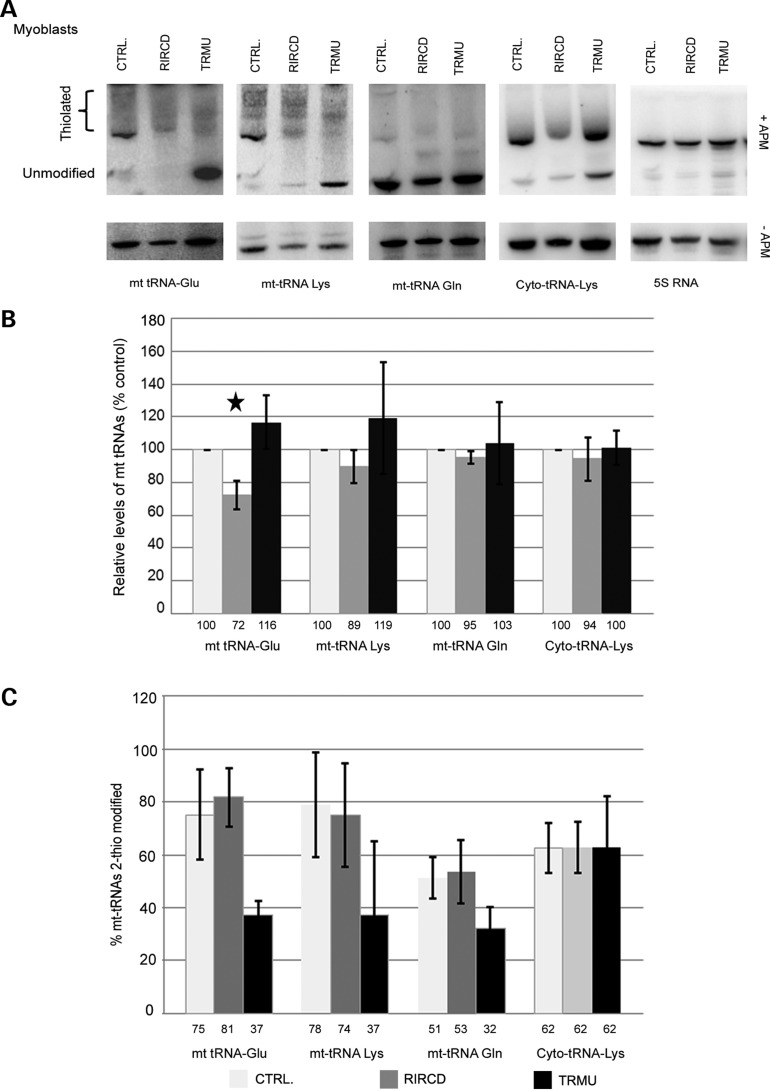
Analyses of 2-thiouridine modification of mt-tRNA species in RIRCD, TRMU and control myoblasts. APM, (*N*-)acroylamino-phenyl-mercuric chloride); RIRCD, reversible infantile respiratory chain deficiency; TRMU, patient cells carrying the TRMU mutation; CTRL, control. (**A**) Northern blotting with adding APM to the gels to separate thiolated and unthiolated tRNA species was performed and probed for mt-tRNA^Glu^, mt-tRNA^Lys^, mt-trNA^Gln^, cytoplasmic tRNA^Lys^ and 5S rRNA in immortalized human myoblasts of patients with RIRCD, TRMU deficiency and control cell lines. Results derive from two independent experiments, all representative blots were used for all tRNA probes following each other. (**B**) Quantification of the northern blots shows relative steady-state levels of the tRNAs and (**C**) the percentage of thiolated tRNA species compared with the whole amount of each tRNAs. For each sample the signal corresponding to the amount of tRNA was normalized to the signal corresponding to the amount of 5S RNA. The total levels of each of the four thio-modified tRNAs in the control cells were set arbitrarily to 100%. The values in the histogram are averages of two measurements, one corresponding to the signal from the gel without APM and the other to the total signal (thiolated plus unmodified) from the gel containing APM. The quantification of the modification is presented at the bottom panel and is expressed as a percentage of the thiolated signal from the thiolated + non-thiolated signals.

The relative steady-state level of mt-tRNA^Glu^ was reduced in both myoblasts (Fig. 1A and B) and fibroblasts (Supplementary Material, Fig. S1A and B) from RIRCD patients, as shown previously (3). Steady-state levels of mt-tRNA^Lys^ and mt-tRNA^Gln^ in RIRCD fibroblasts were also slightly decreased, while this reduction was subtle in myoblasts. The TRMU patient's myoblasts showed an increase in the steady-state level of mt-tRNA^Glu^ and mt-tRNA^Lys^ (Fig. 1A and B), but there was no change of steady-state levels of the three thiolated mt-tRNAs in fibroblasts (Supplementary Material, Fig. S1A and B).

In all cell lines, mt-tRNA^Gln^ was less thiolated than mt-tRNA^Glu^ and mt-tRNA^Lys^. While TRMU-deficient fibroblasts and myoblasts showed defective thiolation of mt-tRNA^Glu^ and mt-tRNA^Lys^ thiolation (Fig. 1C, Supplementary Material, Fig. S1C) in RIRCD-deficient cells was similar to controls (Fig. 1C, Supplementary Material, Fig. S1C).

### Down-regulation of TRMU (siRNA) decreased 2-thiouridylation and steady-state level of mt-tRNA^Glu^ in RIRCD patient cells

**Figure 2.**
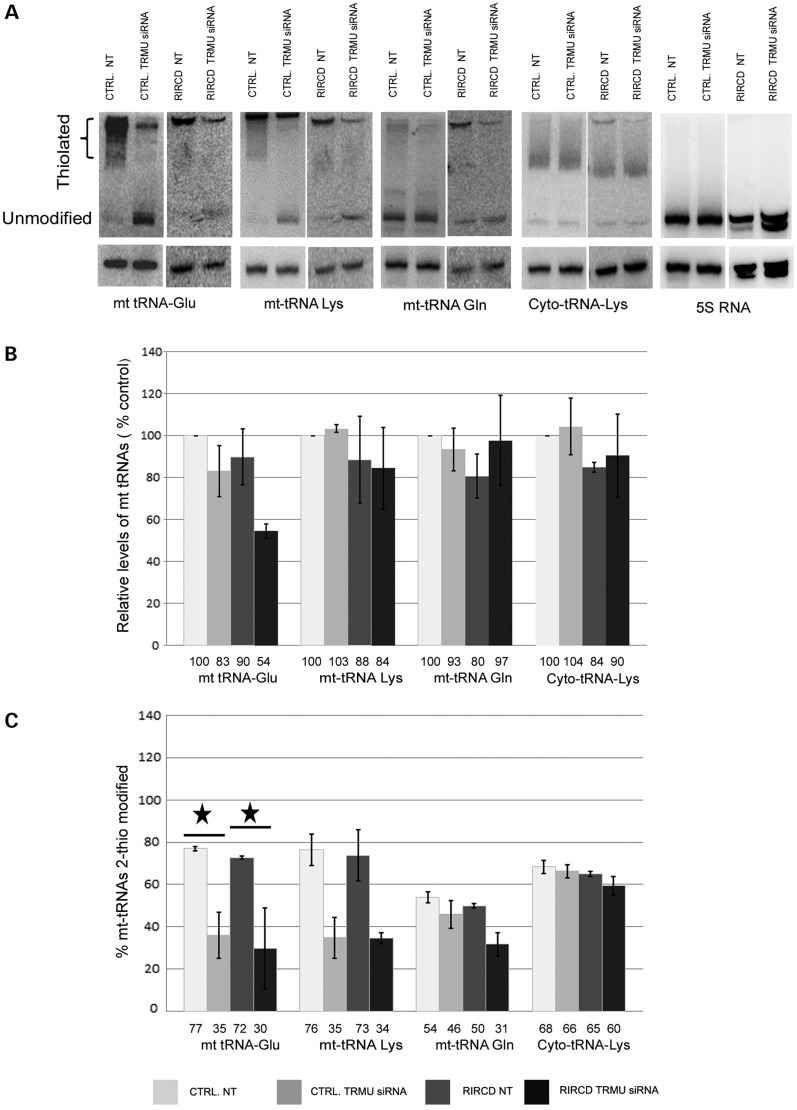
Ablation of TRMU decreased 2-thiouridylation and steady-state level of mt-tRNA^Glu^ in RIRCD patient myoblasts. (**A**) Northern blotting with/without APM was performed in RIRCD and control cells after down-regulation of TRMU by siRNA or treatment by non-targeting siRNA (NT). Results derive from the same experiment; blots were used for all tRNA probes subsequently. Representative northern blots were quantified as described in Figure 1. (**B**) Relative steady-state levels of the tRNAs. (**C**) We show the percentage of thiolated tRNA species compared with the whole amount of each tRNAs in the studied cell lines.

To investigate whether an additional impairment of 2-thiouridylation compromises the mitochondrial translation defect in RIRCD, we down-regulated TRMU in fibroblasts and myoblasts of a patient. We used the siRNA, which showed the most prominent decrease of TRMU protein on immunoblotting (11). After siRNA-mediated down-regulation, both RIRCD and control fibroblasts (Supplementary Material, Fig. S2C) and myoblasts (Fig. 2C) showed low levels of thiolation of mt-tRNA^Glu^ and mt-tRNA^Lys^, when compared with treatment with non-targeting siRNA (NT). Thiolation of mt-tRNA^Gln^ was low before siRNA treatment and down-regulation of TRMU caused only a minor change in both patient and control (Fig. 2C). Cytoplasmic tRNAs were not fully thiolated; however, down-regulation of TRMU did not alter thiouridylation of cytoplasmic tRNAs (Fig. 2C). A part of mt-tRNA^Glu^, mt-tRNA^Lys^ and mt-tRNA^Gln^ always remained unthiolated. Down-regulation of TRMU further significantly compromised the steady-state level of tRNA^Glu^ in RIRCD myoblasts compared with non-targeting siRNA-treated cells (Fig. 2A and B).

### Down-regulation of TRMU impaired mitochondrial translation in RIRCD myoblasts

**Figure 3.**
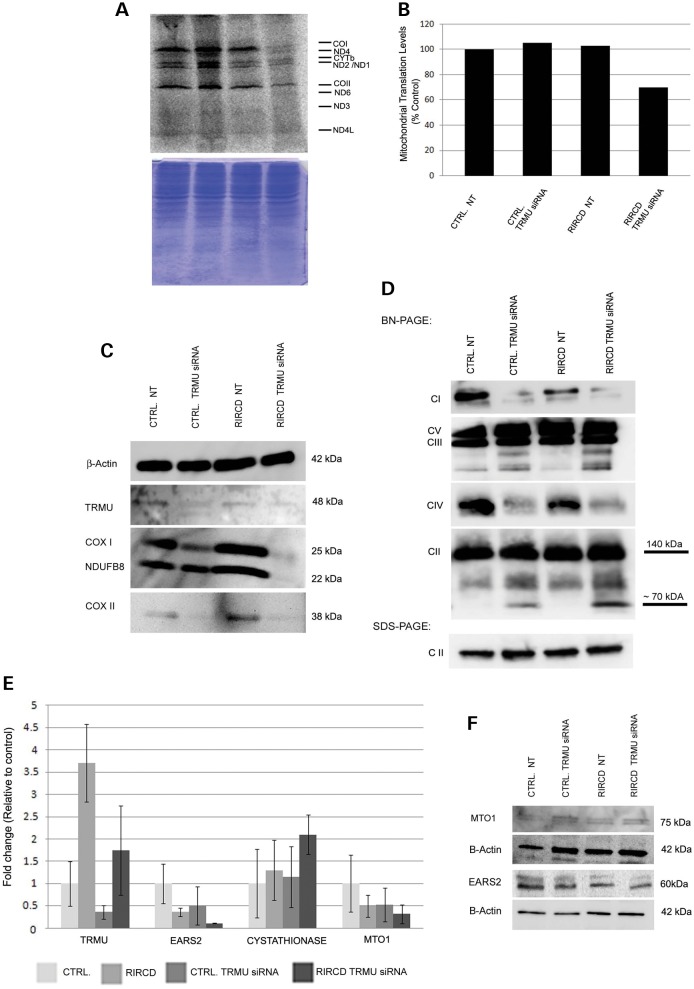
Down-regulation of TRMU hinders mitochondrial protein translation, protein synthesis and modifies the gene expression of other mt-tRNA modifier enzymes. (**A**) ^35^S-Methionine pulse labelling for mitochondrial translation after down-regulation of TRMU resulted in a decreased mitochondrial translation in RIRCD cells, but not in controls. (**B**) Histogram of the representative translation assay. NT, non-targeting siRNA. (**C**) Immunoblotting detected very low mitochondrial protein levels for COX I, COX II and NDUFB8 after down-regulation of TRMU in RIRCD cells. TRMU depletion resulted in mildly decreased COX I and COX II and normal NDUFB8 in controls. β-Actin was used as a loading control. (**D**) Blue native PAGE detected decreased complex I and IV in RIRCD myoblasts, and a mild decrease of complex I and IV in controls after down-regulation of TRMU by siRNA. Complex II showed an additional band if TRMU was down-regulated both in RIRCD cells and controls. Complex III was normal, but we also detected some additional bands by complex V antibody. Immunoblotting with complex II antibodies was used as a loading control. (**E**) Real-time PCR analysis indicated elevated gene expression of TRMU in RIRCD myoblasts compared with control cells and following TRMU siRNA transfection the gene expression, as expected, decreased in both cell lines. The expression of EARS2 and the MTO1 (another tRNA modifying enzyme) seemed to be lower in the patient cell line when comparing to control and this further decreased after TRMU down-regulation. The Cystathionase (*CST*) expression, however, increased after the siRNA transfection. (*n* = 3). Data are represented as the mean ± SD. (**F**) Immunoblotting for MTO1 and EARS2 in the same cell lines detected no significant change in protein expressions.

We reported previously that mitochondrial translation is normal in both fibroblasts and myoblasts of RIRCD patients studied by ^35^S-methionine pulse labelling (3). However, down-regulation of TRMU by siRNA resulted in an impairment of mitochondrial translation in RIRCD myoblasts, while mitochondrial translation in controls was slightly increased, perhaps indicating a compensatory mechanism (Fig. 3A and B).

### Down-regulation of TRMU decreased mitochondrial protein levels in RIRCD myoblasts

Down-regulation of TRMU in RIRCD myoblasts resulted in a severe decrease of protein levels of the mitochondrial complex IV subunits COX I, COX II and also for NDUFB8, representing mitochondrial complex I subunits (Fig. 3C). Control cells showed mildly decreased steady-state levels of COX I, COX II and no change was observed in NDUFB8 (Fig. 3C).

### Blue native polyacrylamide gel electrophoresis (BN-PAGE) and in-gel activity of oxidative phosphorylation complexes

BN-PAGE and ‘in gel’ activity measurement detected slightly reduced complex I and IV in untreated RIRCD myoblasts compared with controls, and this was the only ‘cellular phenotype’ of a defective mitochondrial translation (Fig. 3D). Down-regulation of TRMU resulted in a further decrease of complex I and IV in RIRCD cells, but also led to a decrease in controls. There was an additional ∼70 kDa complex II intermediate noted in TRMU down-regulated cells, similarly to previously reported data in TRMU-deficient human primary fibroblasts (11). Complex III remained unchanged. In addition, non-specific complex V assembly intermediates were detected in TRMU down-regulated cells, which we consider to be a non-specific finding (Fig. 3D).

### Thiolation of mt-tRNA^Glu^ or the m.14674T>C mutation may affect *EARS2* and *MTO1* gene expression

To explore potential compensatory mechanisms, we studied the effects of TRMU siRNA on the glutamyl-tRNA synthetase (EARS2), and MTO1, another enzyme affecting the 5-carboxymethylamino-methylation of the same nucleotide (U34) of the wobble position of mt-tRNA^Glu^, mt-tRNA^Gln^ and mt-tRNA^Lys^. Gene expression levels of both *EARS2* and *MTO1* were reduced in RIRCD compared with controls, and further decrease was detected after TRMU depletion, suggesting that deficient 2-thiolation may alter these other important mt-tRNA^Glu^ modifying factors (Fig. 3E). Depletion of TRMU resulted in increased expression of the gene-encoding cystathionase, the enzyme responsible for cysteine production. The decreased *EARS2* and *MTO1* gene expression did not result in significant protein reduction, possibly due to the short period of the siRNA experiment (Fig. 3F).

Importantly, the higher level of *MTU1* gene expression in the RIRCD patient cells may be a compensatory change which further confirms the link between the two reversible mitochondrial conditions. Moreover, RT–PCR of skeletal muscle of a TRMU patient and early muscle biopsy of an RIRCD patient showed significantly higher *MTU1* gene expression, which decreased in parallel with clinical recovery in a follow-up muscle.

### Investigation of 2-thiouridylation in control and patient skeletal muscle

While steady-state levels of all mt-tRNAs, but not the cytoplasmic tRNA^Lys^, increased gradually by age in human skeletal muscle (Fig. 4A, B and D), the rate of thiolated/non-thiolated tRNA species for mt-tRNA^Lys^, mt-tRNA^Glu^ and mt-tRNA^Gln^ showed no change by age in normal skeletal muscle (Fig. 4C and E). Skeletal muscle of a patient with TRMU deficiency showed impaired thiolation, but slightly increased mt-tRNA steady states, most likely reflecting compensation, suggesting that TRMU defect is not restricted to liver (Fig. 4E).

**Figure 4.**
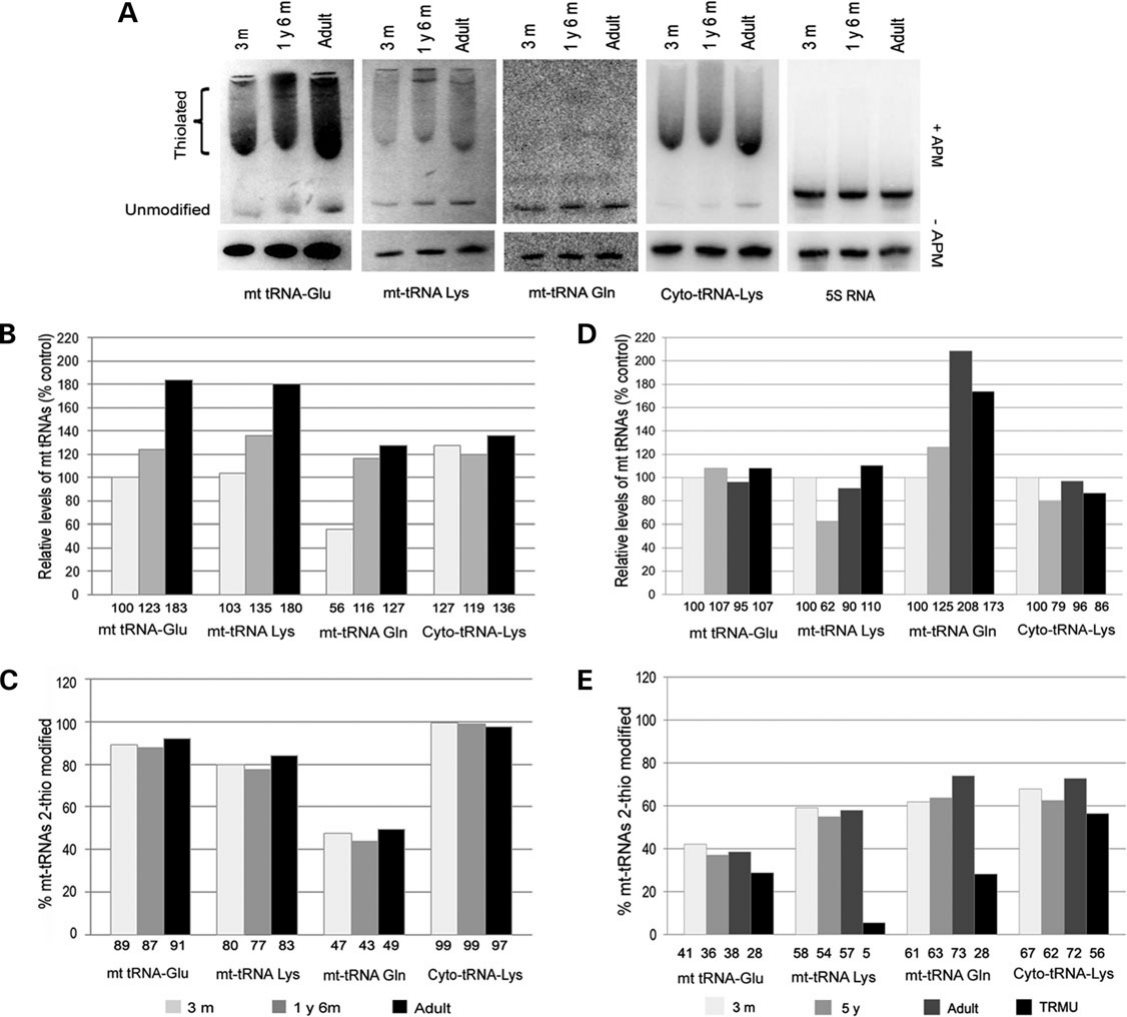
2-Thiouridylation is decreased in RIRCD skeletal muscle. We performed northern blotting in skeletal muscle and probed for mt-tRNA^Glu^, mt-tRNA^Lys^, mt-tRNA^Gln^, cytoplasmic tRNA^Lys^ and 5S rRNA. Quantification of the northern blot results shows the relative steady state and percentage of thiolated tRNA species compared with the whole amount of each tRNAs. (**A**) Northern blotting with and without APM has been performed in skeletal muscle of control individuals of different age. 3 m, 3 month; 1y 6 m, 1 year 6 months. Representative blots were used for all tRNA probes following each other. (**B** and **C**) Quantification of the representative northern blot results shows the relative steady state and percentage of thiolated tRNA species compared with the whole amount of each tRNAs. (**D** and **E**) Northern blotting with and without APM has also been performed in skeletal muscle of other control individuals of different age and in a TRMU patient (only quantification is shown). (**F–K**) Quantification of the northern blot results (with and without APM) was performed in skeletal muscle of follow-up biopsies of two RIRCD patients in the symptomatic phase and after recovery. (F–H) Relative steady-state and percentage of thiolated tRNA species compared with the whole amount of each tRNAs in Patient 1. Quantification of the northern blot results shows the relative steady state and percentage of thiolated tRNA species compared with the whole amount of each tRNAs. (**I–K**) Relative steady state and percentage of thiolated tRNA species compared with the whole amount of each tRNAs in Patient 2.

**Figure 4.**
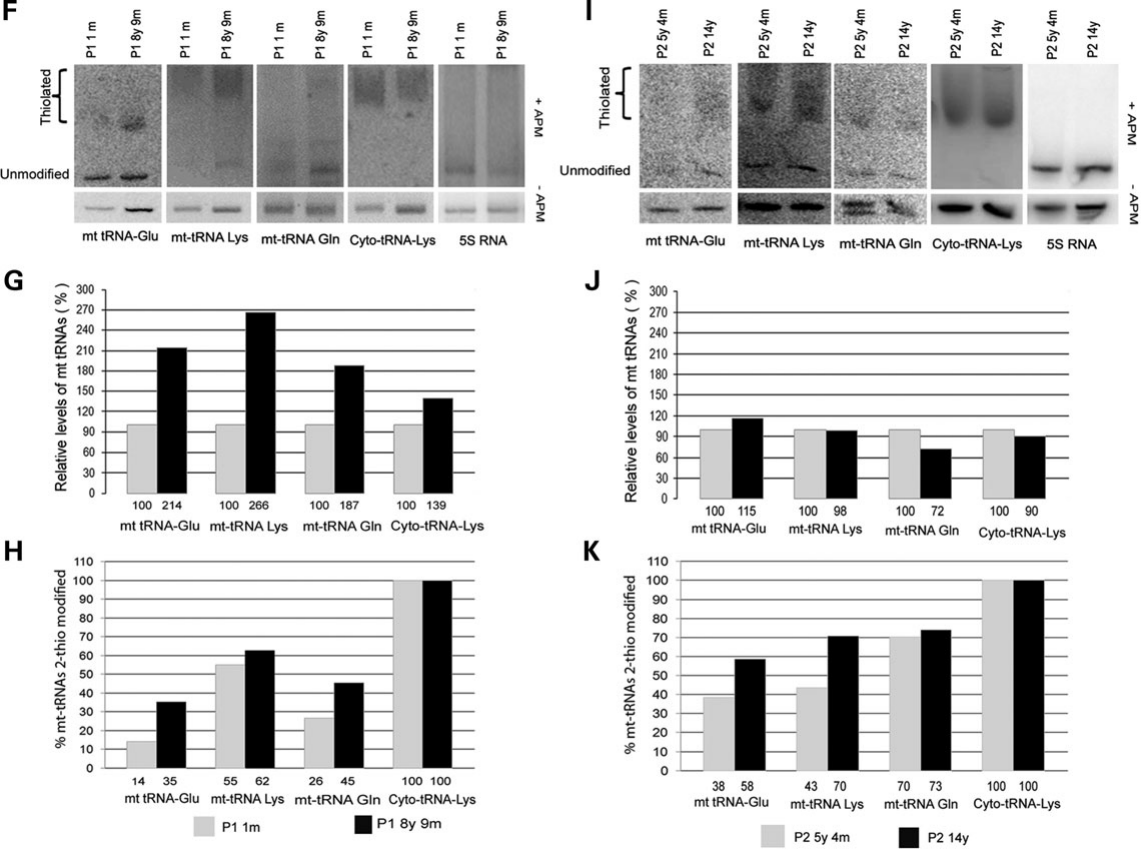
Continued

Follow-up skeletal muscle biopsies of two previously reported RIRCD patients (3) were studied and showed very low levels of thiolated mt-tRNA^Glu^, and also slightly lower levels of mt-tRNA^Lys^ and mt-tRNA^Gln^ in the symptomatic phase of the disease (1 months, 5 years 4 months of age) (Fig. 4F, I and H, K). The second patient had an unusually long symptomatic phase causing symptoms until at least 7 years of age. There was a 20% increase in the thiolated mt-tRNA^Glu^ levels in both patients between early (1 month, 5 years 4 months), and follow-up biopsies after clinical recovery (8 years 9 months and 14 years of age) (Fig. 4H and K). Both mt-tRNA^Lys^ and mt-tRNA^Gln^ showed an increase in thiolation status between the early and late biopsies, while thiolation of the cytoplasmic tRNAs did not change. In addition, mt-tRNA steady states were also lower in the symptomatic phase, further compromising mt-tRNA function (Fig. 4G and J). Repeated analysis was not possible because of the small amount of available skeletal muscle.

### *In vitro* l-cysteine supplementation resulted in improved mitochondrial respiratory function

**Figure 5.**
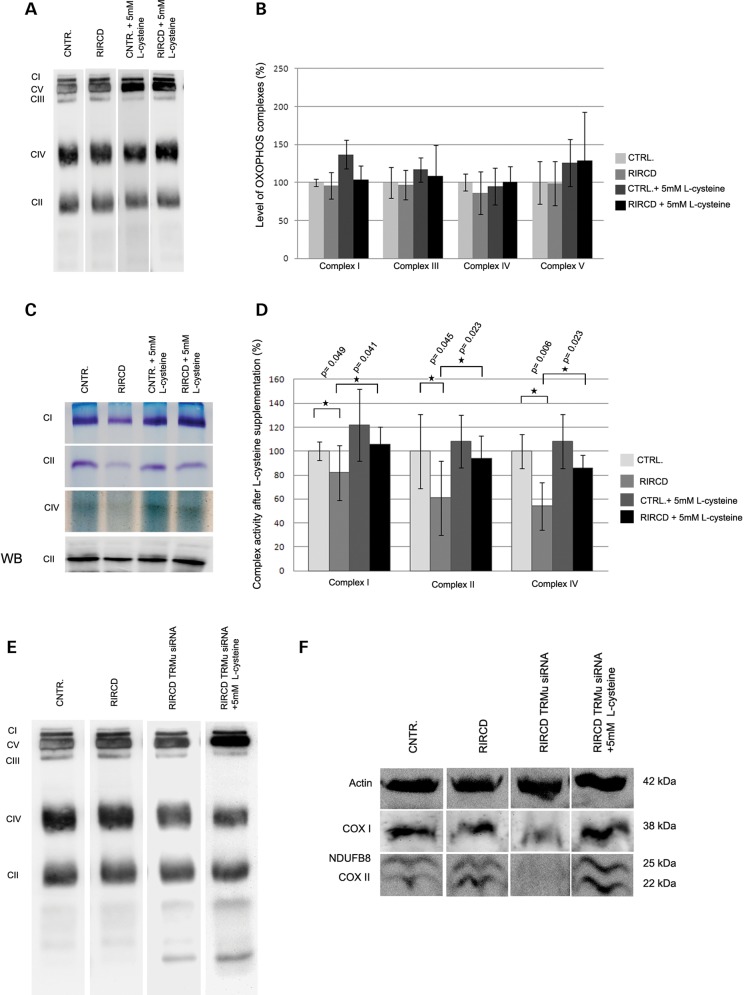
*In vitro* l-cysteine supplementation improved the mitochondrial translation defect and increased complex activities. (**A** and **B**) Blue native PAGE indicated higher level of OXPHOS complexes after l-cysteine supplementation in both control and RIRCD myoblasts (*n* = 5). Data were normalized to the complex II and are presented as the mean ± SD. (**C** and **D**) ‘In gel’ activity measurements also demonstrated significantly increased complex activities in both cell lines after l-cysteine supplementation. SDS–PAGE detection of complex II was used as loading control. (*n* = 5). Data are presented as the mean ± SD. (**E**) Blue native PAGE after TRMU down-regulation in a control and a RIRCD cell line. (**F**) Immunoblotting after TRMU down-regulation in an RIRCD cell line, 5 mm l-cysteine prevented the mitochondrial translation defect of mitochondrial proteins (Complex I and Complex IV) when TRMU was down-regulated in RIRCD cells.

**Figure 6.**
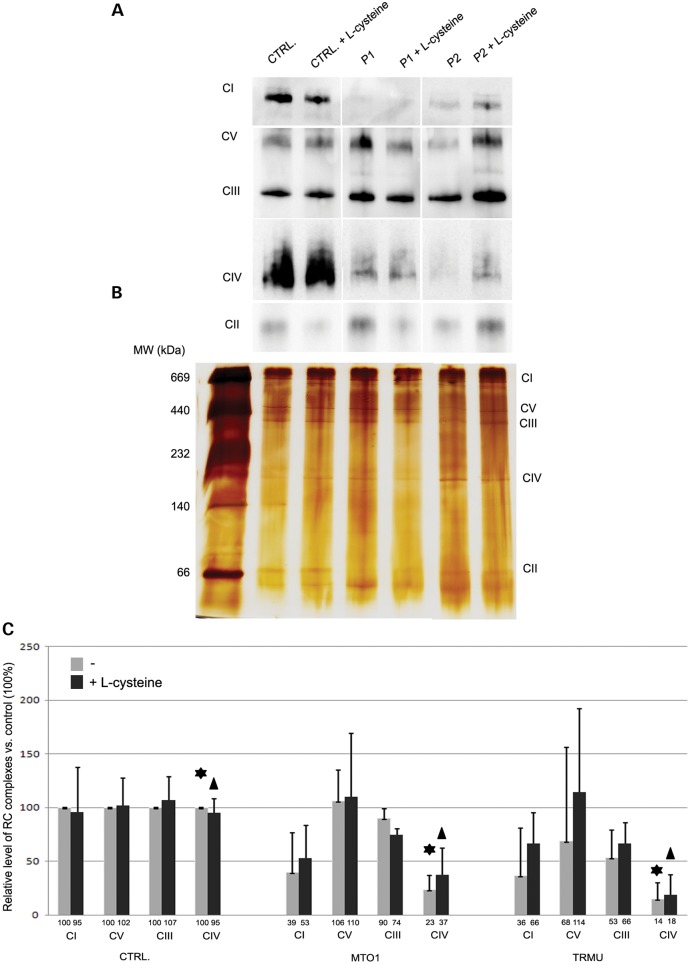
*In vitro* l-cysteine supplementation increased the level of mitochondrial complexes in both TRMU- and MTO1-deficient fibroblasts. (**A**) Blue native PAGE indicated low level of complex I and IV in both patient cell lines compared to control fibroblasts. l-cysteine treatment improved the low level of these RC complexes. (**B**) Silver-stained mitochondrial complexes shown as loading control before and after treatment. (**C**) The relative level of RC complexes compared with control cells. Data were normalized to the complex II and are presented as the mean ± SD (*n* = 3). The control value obtained for the control untreated fibroblasts was represented as 100% and the value from the l-cysteine treated cells was expressed as a percentage of the control value. The asterisk denotes that the level of complex IV was significantly lower in the MTO1 and TRMU patient cells compared to control (*P* ≤ 0.004, ANOVA). The triangle indicates the significance after l-cysteine supplementation (*P* ≤ 0.006, ANOVA).

We investigated whether addition of l-cysteine, a substrate of TRMU, required as a source of sulphur for thio-modification, has an effect on TRMU function. *In vitro* supplementation of RIRCD cells with l-cysteine rescued slightly reduced complex I and IV on BN-PAGE (Fig. 5A and B) and significantly increased ‘in gel’ enzyme activities, both in RIRCD and in control myoblasts (Fig. 5C and D). Down-regulation of TRMU resulted in a further decrease of complex I and IV in RIRCD patient cells, and these changes were completely prevented by adding 5 mm l-cysteine to the culture medium (Fig. 5E). The positive effect of l-cysteine on mitochondrial translation was also confirmed by normal immunoblotting of mitochondrial proteins (COX I, COX II and NDUFB8) in RIRCD cells, if l-cysteine was added to the cell culture medium during TRMU down-regulation (Fig. 5F). Cysteine supplementation also improved the respiratory chain enzymes in MTO1- and TRMU-deficient fibroblasts. Our data indicated around a 20 and 30% improvement of complex I and complex IV, respectively, in both patient cell lines. This tendency was more noticeable in TRMU-deficient cells, where all complexes improved after the treatment (Fig. 6A and C).

## DISCUSSION

The synthesis of the 13 mitochondrial-encoded proteins is a complex pathway, which requires ∼150 different proteins (ribosomal proteins, ribosomal assembly proteins, aminoacyl-tRNA synthetases, tRNA-modifying enzymes, tRNA methylating enzymes and several initiation, elongation and termination factors) involved in mitochondrial translation (12–14). Most of these gene defects result in histological (COX-deficient or ragged red fibres) and biochemical abnormalities (multiple respiratory chain defects) in affected organs. The clinical phenotypes are usually early-onset, severe and often fatal, implying the importance of mitochondrial translation from birth (15). Some of these conditions affect multiple tissues; however, tissue-specific manifestations have been reported for several mt-tRNA aminoacyl synthetases or mt-tRNA-modifying genes (13,14).

Based on the striking similarities between two clearly reversible mitochondrial conditions, RIRCD due to a homoplasmic mt-tRNA^Glu^ mutation and reversible infantile liver failure due to TRMU deficiency, we hypothesized that the reversibility may be due to basic mechanisms involving mt-tRNA^Glu^. The importance of mt-tRNA^Glu^ in reversible disease is also supported by the partial recovery of patients with mutations in other two recently identified mt-tRNA^Glu^-modifying genes (*EARS2* and *MTO1*) (9,10). Reversibility (or even improvement) is an extremely rare event in severe childhood mitochondrial disorders and 2-thiouridylation may offer a common pathway; therefore, we studied whether modifying thiolation of the U34 position on mt-tRNA^Glu^, which is affected in reversible infantile liver failure, could contribute to the muscle-specific RIRCD, caused by the homoplasmic m.14674T>C/G mutation in mt-tRNA^Glu^.

The uridine at the first anticodon position (U34, wobble position) is present only in the anticodon of 3 mt-tRNAs (Glu, Lys and Gln). It is modified at carbons 2 and 5, and while carbon 2 is modified exclusively through thiolation (s^2^), various methyl derivates can be found at carbon 5 (methylaminomethyl mmm^5^, carboxymethylaminiomethyl cmmm^5^, etc.) (16). The 2-thio group is required for the efficient codon recognition, and in the case of mt-tRNA^Glu^, it is necessary for the recognition by the glutaminyl-tRNA synthetase (17). The 2-thio group confers conformational rigidity, ensuring stable and accurate codon–anticodon pairing, and causes a steric repulsion with its 2′ OH group at the 3′ end of the tRNA, therefore an interaction between these two positions may be possible (17). *Vice versa*, an altered 2-thiouridylation may further impair the mutant but still functioning mt-tRNA^Glu^, possibly similar to its effect in the case of m.1555A>G (18).

We detected minor changes of 2-thiouridylation of mt-tRNA^Glu^ in RIRCD fibroblasts and no change in myoblasts, suggesting that the homoplasmic m.14674T>C/G mutation *per se* does not affect thiolation of mt-tRNA^Glu^. To further explore the effect of an impaired 2-thiouridylation on function of the mutant mt-tRNA^Glu^, we depleted TRMU in primary patient cells *in vitro.* Down-regulation of TRMU resulted in defective 2-thiouridylation of all 2-thiolated mt-tRNAs (mt-tRNA^Lys^, mt-tRNA^Glu^, mt-tRNA^Gln^) in both fibroblasts and myoblasts of a patient with RIRCD, as well as in controls. Importantly, the impairment of 2-thiouridylation of mt-tRNA^Glu^ was most severe in RIRCD myoblasts, implicating that mutant m.14674T>C myoblasts are more sensitive for the 2-thiouridylation defect of mt-tRNA^Glu^, triggered by the depletion of TRMU, than RIRCD fibroblasts or control myoblasts.

It was suggested previously that TRMU is not required for mitochondrial translation if steady-state levels of mt-tRNAs are normal (11). Furthermore, down-regulation of TRMU did not result in a further impairment of mitochondrial translation in fibroblasts or myoblasts carrying the m.3243A>G (MELAS) and m.8344A>G (MERRF) mutations (11). Our results are supporting these previous studies that depletion of TRMU did not significantly alter mitochondrial translation on pulse labelling in controls, however, unlike in MELAS and MERRF myoblasts, down-regulation of TRMU resulted in an impaired mitochondrial protein synthesis in RIRCD myoblasts. This was further confirmed by a severe decrease of mitochondrial subunits (immunoblotting) and assembled complexes (BN-PAGE). The reasons behind these differences can be mutation specific, or other functions of TRMU may be involved (11). An as yet uncharacterized function of TRMU in sulphur-trafficking was suggested previously (11). However, a defect in iron–sulphur (Fe–S) biosynthesis would not affect COX, which does not contain an Fe–S centre, therefore cannot explain the full biochemical phenotype caused by down-regulation of TRMU.

It was suggested before that the 2-thiouridylation of mt-tRNA^Glu^ affects not only the accuracy and efficiency of translation, but also important for the recognition of the tRNA by the mitochondrial glutamyl-tRNA synthetase (EARS2). A disturbance of this interaction, possibly altered by the m.14674T>C/G mutation, would further contribute to the defect in mitochondrial translation in RIRCD (19). In support of this hypothesis, in RIRCD cells gene expression of *EARS2* was lower than in controls and down-regulation of TRMU resulted in a further decrease of *EARS2* gene expression. TRMU down-regulation also led to decreased *EARS2* expression in controls, suggesting that thiolation may affect other mt-tRNA^Glu^ modifications.

A synergistic effect of the yeast proteins involved in 2-thiouridylation (MTU1) and methylaminomethylation (MTO1) of the U34 wobble nucleotide was suggested previously (19,20). We studied whether down-regulation of the 2-thiouridylation alters MTO1 in our cellular model and similar to *EARS2*, *MTO1* expression was decreased in RIRCD myoblasts. TRMU down-regulation resulted in a further decrease in *MTO1* gene expression in RIRCD and also in control myoblasts, suggesting a link between the two modification steps of U34.

To explain the age-dependent, tissue-specific infantile presentation of reversible mitochondrial disease, we studied physiological or developmentally regulated changes in 2-thiouridylation in skeletal muscle biopsies of patients of different age. Our results suggest that the level of thiolated and non-thiolated tRNA species in normal human skeletal muscle does not change by age; however, steady-state levels of mt-tRNAs increase during the first years of life. Most importantly, skeletal muscle of two RIRCD patients in the symptomatic phase showed clearly decreased thiolation and mt-tRNA steady-state levels which improved in parallel with the clinical recovery, providing experimental evidence for a role of thiolation in the reversibility.

The most exciting result of our study was the effect of *in vitro* l-cysteine supplementation. BN-PAGE showed minor abnormalities in RIRCD myoblasts, similar to a previous study (4), and a defect of complexes I and IV was more pronounced on the ‘in gel’ activity assay. Adding l-cysteine to the culture medium fully reversed this deficiency. Furthermore, l-cysteine prevented the decrease in respiratory complexes in TRMU down-regulated RIRCD cells and controls, supporting that low cysteine concentrations may play a role in triggering a reversible mitochondrial translation defect *in vitro*, and this can be rescued by l-cysteine supplementation. l-Cysteine supplementation led to an improvement in most respiratory chain complex activities in TRMU- and MTO1-deficient cells, indicating that the positive effect is not specific to the thio-modification.

Recent publications suggested a possible beneficial effect of supplementation with *N*-acetylcysteine, a precursor of sulphide-buffering glutathione in mice and patients with a rare mitochondrial condition, ethylmalonic encephalopathy due to mutations in the *ETHE1* gene encoding a mitochondrial sulphur dioxygenase (21). A double-blind cross-over study on patients with mitochondrial myopathies showed that 30-day supplementation with a whey-based cysteine donor resulted in significantly reduced oxidative stress (22), and a recent paper reported lower levels of reduced cysteine and thiols in plasma of children with mitochondrial diseases, suggesting that relative thiol deficiency could be an important factor in paediatric mitochondrial conditions (23).

**Figure 7.**
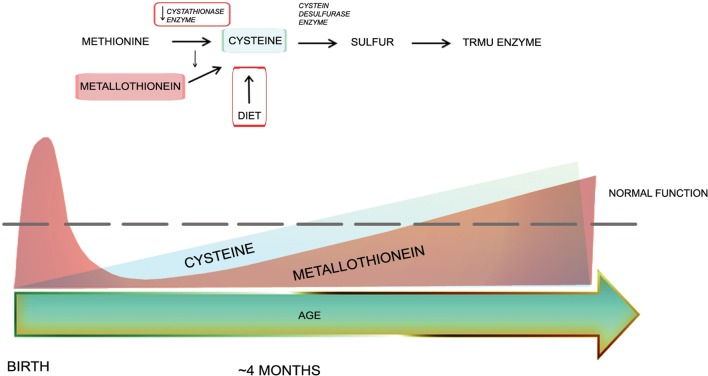
Schematic representation of cysteine sources for functional TRMU enzyme. The cystathionase enzyme or also called cystathionine gamma-lyase plays an essential role in cysteine production. However, in the early months of life the activity of this enzyme is low. Metallothionein which represents another cysteine source, although presents at high levels at birth, dramatically decreases in the neonatal period. Therefore, the production of this amino acid is limited. Dietary cysteine intake might play a crucial role for the normal TRMU enzyme activity within the first few months of life when combined with underlying genetic diseases.

How does cysteine play a role in reversible mitochondrial disease in infants? TRMU protein requires sulphur for its activity supplied by the cysteine desulfurase enzyme. Since the availability of cysteine in the neonatal period is limited by the low activity of the cystathionase enzyme, dietary cysteine intake may be very important at this age. It was hypothesized that between 1 and 4 months of age inter-current illnesses, combined with reduced dietary cysteine intake, may compromise TRMU activity, resulting in decreased 2-thiolation (7). Decreased cysteine levels could reflect differences in nutrition, or could be due to other environmental, genetic or epigenetic factors (Fig. 7). Our data suggest that l-cysteine supplementation may potentially reverse the age-dependent clinical manifestation of RIRCD and TRMU deficiency. Further investigation of infantile cysteine levels may help to unveil these mechanisms which can have important implications in reversible mitochondrial disease, but also in other mitochondrial conditions.

## MATERIALS AND METHODS

### Cell culture and siRNA transfection

Fibroblast and myoblast cell cultures of two RIRCD patients, a TRMU-deficient and a MTO1-deficient cell line as well as controls (Supplementary Material) were obtained from the Biobank of the Medical Research Council, Centre for Neuromuscular Diseases, Newcastle, and were immortalized as described previously (24). Informed consent was obtained from all subjects. Fibroblasts were grown in high glucose Dulbeccos modified Eagle's medium (Sigma, Poole, UK) supplemented with 10% foetal bovine serum. Muscle cells were grown in skeletal muscle growth medium (PromoCell, Heidelberg, Germany), supplemented with 4 mm l-glutamine and 10% foetal bovine serum and cultured as recommended by the supplier. Stealth RNAi duplexes (TRMU HSS124809 or HSS124809 siRNA) were transiently transfected at a final concentration of 12 nm using Lipofectamine RNAiMAX (invitrogen), according to the manufacturer's specifications. Transfections were repeated on Day 3, cells were either harvested or again transfected on Day 6, with cells being harvested on Day 9.

### Supplementation with l-cysteine

Myoblasts were grown in skeletal muscle growth medium (described above, 0.2 mm l-cysteine), supplemented with 5 mm l-cysteine (Sigma). Cells were left to grow for 5–9 days. The medium was changed every 72 h.

### Immunoblotting

For immunoblotting, protein extracts were prepared as described previously (15). Aliquots of total protein (5–20 μg) were loaded on 14% sodium dodecyl sulphate–polyacrylamide gels (SDS–PAGE), transferred to polyvinylidene fluoride membranes and subsequently used for detection of TRMU, with a polyclonal, affinity purified antibody (from Prof. E. Shoubridge) at a dilution of 1:1000. The blots were also probed with monoclonal antibodies recognizing mitochondrial COX I (Molecular Probes), COX II (Mitosciences) or NDUFB8 (Mitosciences), EARS2 (Abgent), MTO1 (Proteintech Group, Inc.) and β-actin (Sigma) according to the recommendations of the suppliers.

### APM-northern blotting analysis

Isolation of RNA from both cells and tissues was carried out using Trizol® (Invitrogen) following the manufacturers recommendations. We performed northern blotting on APM containing gels, essentially using the method described previously (11). This is the standard method to separate thiolated and non-thiolated tRNA species (16). Following transfer to GeneScreen Plus membrane (Perkin Elmer), the presence of tRNA species was detected using ^32^P-labelled PCR products as described previously (25). The probes for human mt-tRNA^Glu^, mt-tRNA^Lys^, mt-tRNA^Gln^, the cytoplasmic tRNA^Lys^ and 5S RNA were generated using primers listed in the Supplementary material. Quantification of the radioactive signal was performed with imageJ software.

### Pulse-labelling of mitochondrial translation products

*In vivo* ^35^S-metabolic labelling studies were performed as described previously (11,26) with the following modifications. Cells, cultured to 60–70% confluency in T25 mm flasks, washed with phosphate-buffered saline (PBS; Sigma) and washed by incubating twice for 10 min at 37°C/5% CO_2_ in methionine/cysteine-free DMEM (Sigma, Poole, UK), with the media replaced between each incubation. Cells were then incubated for 15 min at 37°C/5% CO_2_ in methionine/cysteine-free DMEM supplemented with 5% (v/v) dialyzed FBS, 0.1 mg/ml emetine dihydrochloride (Sigma). Following addition of 200 mCi/ml ^35^S-methionine/cysteine (^35^S EasyTag EXPRESS; Perkin Elmer), cells were incubated for 15 min at 37° C/5% CO_2_, then washed twice with ice-cold DMEM supplemented with 7.5 mg/ml methionine. Cell pellets were prepared after washing once with ice-cold PBS. Radio-labelled proteins were then analyzed using SDS–PAGE as described previously (3).

### RT–PCR

RNA was isolated from myoblasts after non-targeted and siRNA transfection (Arcturus PicoPure RNA isolation kit; Applied Biosciences). cDNA was prepared using 0.5 μg RNA and RT–PCR was performed with SYBR Green detection. Data were normalized to β-actin and evaluated by ΔΔCt and standard curve analysis. Melting curves from PCR products showed a single peak and product sizes were confirmed with gel electrophoresis. Primer sequences used in RT–PCR reactions for human *TRMU*, *EARS2*, *MTO1*, cystathionase (*CTH*), and β-actin (*ACTB*) are listed in the Supplementary Material.

### Blue native poly-acrylamide gel electrophoresis (BN-PAGE) and ‘in gel’ activity

BN-PAGE has been performed as described previously (27). After electrophoresis activities, ‘in gel’ assays were carried out as described previously (28).

### DNA analysis

Direct sequencing of TRMU has been performed and showed no pathogenic variants in DNA extracted from the analyzed cell lines and in five affected and five unaffected family members by standard methods (9).

### Statistical analysis

The statistical package SigmaPlot 11.0 was used to perform all the statistics. Data are presented as mean ± standard deviation. The normal distribution was checked by the Kolmogorov–Smirnov test and ANOVA tests were used to compare parameters. The Holm–Sidak method was used for pairwise multiple comparison procedures. A *P*-value of <0.05 was considered significant.

### Image processing

After northern blot analysis, data were quantified using ImageJ software and total RNA levels were corrected for 5S RNA.

## SUPPLEMENTARY MATERIAL

Supplementary Material is available at *HMG* online.

## FUNDING

R.H. was supported by the Medical Research Council (UK) (G1000848) and the European Research Council (309548). Funding to pay the Open Access publication charges for this article was provided by the Wellcome Trust Centre for Mitochondrial Research.

## Supplementary Material

Supplementary Data

## References

[1] Tuppen H.A., Blakely E.L., Turnbull D.M., Taylor R.W. (2010). Mitochondrial DNA mutations and human disease. Biochim. Biophys. Acta.

[2] Ylikallio E., Suomalainen A. (2012). Mechanisms of mitochondrial diseases. Ann. Med..

[3] Horvath R., Kemp J.P., Tuppen H.A., Hudson G., Oldfors A., Marie S.K., Moslemi A.R., Servidei S., Holme E., Shanske S. (2009). Molecular basis of infantile reversible cytochrome c oxidase deficiency myopathy. Brain.

[4] Mimaki M., Hatakeyama H., Komaki H., Yokoyama M., Arai H., Kirino Y., Suzuki T., Nishino I., Nonaka I., Goto Y. (2010). Reversible infantile respiratory chain deficiency: a clinical and molecular study. Ann. Neurol..

[5] Uusimaa J., Jungbluth H., Fratter C., Crisponi G., Feng L., Zeviani M., Hughes I., Treacy E.P., Birks J., Brown G.K. (2011). Reversible infantile respiratory chain deficiency is a unique, genetically heterogeneous mitochondrial disease. J. Med. Genet..

[6] Sitarz K.S., Chinnery P.F., Yu-Wai-Man P. (2012). Disorders of the optic nerve in mitochondrial cytopathies: new ideas on pathogenesis and therapeutic targets. *Curr. Neurol. Neurosci. Rep.*.

[7] Zeharia A., Shaag A., Pappo O., Mager-Heckel A.M., Saada A., Beinat M., Karicheva O., Mandel H., Ofek N., Segel R., Marom D. (2009). Acute infantile liver failure due to mutations in the TRMU gene. Am. J. Hum. Genet..

[8] Schara U., von Kleist-Retzow J.C., Lainka E., Gerner P., Pyle A., Smith P.M., Lochmüller H., Czermin B., Abicht A., Holinski-Feder E. (2011). Acute liver failure with subsequent cirrhosis as the primary manifestation of TRMU mutations. J. Inherit. Metab. Dis..

[9] Ghezzi D., Baruffini E., Haack T.B., Invernizzi F., Melchionda L., Dallabona C., Strom T.M., Parini R., Burlina A.B., Meitinger T. (2012). Mutations of the mitochondrial-tRNA modifier MTO1 cause hypertrophic cardiomyopathy and lactic acidosis. Am. J. Hum. Genet..

[10] Steenweg M.E., Ghezzi D., Haack T., Abbink T.E., Martinelli D., van Berkel C.G., Bley A., Diogo L., Grillo E., Te Water Naudé J. (2012). Leukoencephalopathy with thalamus and brainstem involvement and high lactate ‘LTBL’ caused by EARS2 mutations. Brain.

[11] Sasarman F., Antonicka H., Horvath R., Shoubridge E.A. (2011). The 2-thiouridylase function of the human MTU1 (TRMU) enzyme is dispensable for mitochondrial translation. Hum. Mol. Genet..

[12] Smits P., Smeitink J., van den Heuvel L. (2010). Mitochondrial translation and beyond: processes implicated in combined oxidative phosphorylation deficiencies. J. Biomed. Biotechnol..

[13] Rötig A. (2011). Human diseases with impaired mitochondrial protein synthesis. Biochim. Biophys. Acta.

[14] Chrzanowska-Lightowlers Z.M., Horvath R., Lightowlers R.N. (2011). 175th ENMC International Workshop: Mitochondrial protein synthesis in health and disease, 25–27th June 2010, Naarden, The Netherlands. Neuromuscul. Disord..

[15] Kemp J.P., Smith P.M., Pyle A., Neeve V.C., Tuppen H.A., Schara U., Talim B., Topaloglu H., Holinski-Feder E., Abicht A. (2011). Nuclear factors involved in mitochondrial translation cause a subgroup of combined respiratory chain deficiency. Brain.

[16] Noma A., Sakaguchi Y., Suzuki T. (2009). Mechanistic characterization of the sulfur-relay system for eukaryotic 2-thiouridine biogenesis at tRNA wobble positions. Nucleic Acids Res..

[17] Madore E., Florentz C., Giegé R., Sekine S., Yokoyama S., Lapointe J. (1999). Effect of modified nucleotides on Escherichia coli tRNAGlu structure and on its aminoacylation by glutamyl-tRNA synthetase. Predominant and distinct roles of the mnm5 and s2 modifications of U34. Eur. J. Biochem..

[18] Guan M.X., Yan Q., Li X., Bykhovskaya Y., Gallo-Teran J., Hajek P., Zhao H., Garrido G., Mengesha E., Suzuki T. (2006). Mutation in TRMU related to transfer RNA modification modulates the phenotypic expression of the deafness-associated mitochondrial 12S ribosomal RNA mutations. Am. J. Hum. Genet..

[19] Umeda N., Suzuki T., Yukawa M., Ohya Y., Shindo H., Watanabe K., Suzuki T. (2005). Mitochondria-specific RNA-modifying enzymes responsible for the biosynthesis of the wobble base in mitochondrial tRNAs. Implications for the molecular pathogenesis of human mitochondrial diseases. J. Biol. Chem..

[20] Wang X., Yan Q., Guan M.X. (2010). Combination of the loss of cmnm5U34 with the lack of s2U34 modifications of tRNALys, tRNAGlu, and tRNAGln altered mitochondrial biogenesis and respiration. J. Mol. Biol..

[21] Viscomi C., Burlina A.B., Dweikat I., Savoiardo M., Lamperti C., Hildebrandt T., Tiranti V., Zeviani M. (2010). Combined treatment with oral metronidazole and *N*-acetylcysteine is effective in ethylmalonic encephalopathy. Nat. Med..

[22] Mancuso M., Orsucci D., Logerfo A., Rocchi A., Petrozzi L., Nesti C., Galetta F., Santoro G., Murri L., Siciliano G. (2010). Oxidative stress biomarkers in mitochondrial myopathies, basally and after cysteine donor supplementation. J. Neurol..

[23] Salmi H., Leonard J.V., Rahman S., Lapatto R. (2012). Plasma thiol status is altered in children with mitochondrial diseases. Scand. J. Clin. Lab. Invest..

[24] Lochmüller H., Johns T., Shoubridge E.A. (1999). Expression of the E6 and E7 genes of human papillomavirus (HPV16) extends the life span of human myoblasts. Exp. Cell. Res..

[25] Taylor R.W., Giordano C., Davidson M.M., d'Amati G., Bain H., Hayes C.M., Leonard H., Barron M.J., Casali C., Santorelli F.M. (2003). A homoplasmic mitochondrial transfer ribonucleic acid mutation as a cause of maternally inherited hypertrophic cardiomyopathy. J. Am. Coll. Cardiol..

[26] Chomyn A. (1996). In vivo labeling and analysis of human mitochondrial translation products. Methods Enzymol..

[27] Leary S.C., Sasarman F. (2009). Oxidative phosphorylation: synthesis of mitochondrially encoded proteins and assembly of individual structural subunits into functional holoenzyme complexes. Methods Mol. Biol..

[28] Diaz F., Barrientos A., Fontanesi F. (2009). Evaluation of the mitochondrial respiratory chain and oxidative phosphorylation system using blue native gel electrophoresis. Curr. Protoc. Hum. Genet..

