# Supplementary material for: Altered 2-thiouridylation impairs mitochondrial translation in reversible infantile respiratory chain deficiency
Source: Hum Mol Genet. 2013 Jun 28;22(22):4602–15. doi: 10.1093/hmg/ddt309 (PMC3889809; doi:10.1093/hmg/ddt309)
Supplement: Supplementary Data [file supp_22_22_4602__index.html]

Altered 2-thiouridylation impairs mitochondrial translation in reversible infantile respiratory chain deficiency — Altered 2-thiouridylation impairs mitochondrial translation in reversible infantile respiratory chain deficiency — Supplementary Data 

# Altered 2-thiouridylation impairs mitochondrial translation in reversible infantile respiratory chain deficiency

## 

Supplementary Data

**Files in this Data Supplement:**

- Supplementary Data - Docx file
