# Supplementary material for: Altered 2-thiouridylation impairs mitochondrial translation in reversible infantile respiratory chain deficiency
Source: Hum Mol Genet. 2013 Jun 28;22(22):4602–15. doi: 10.1093/hmg/ddt309 (PMC3889809; doi:10.1093/hmg/ddt309)
Supplement: Supplementary Data [file supp_ddt309_ddt309supp.docx]

**SUPPLEMENTARY MATERIAL**

**Supplementary Figure 1**

Analyses of 2-thiouridine modification of mt-tRNA species in RIRCD, TRMU and control cell lines.


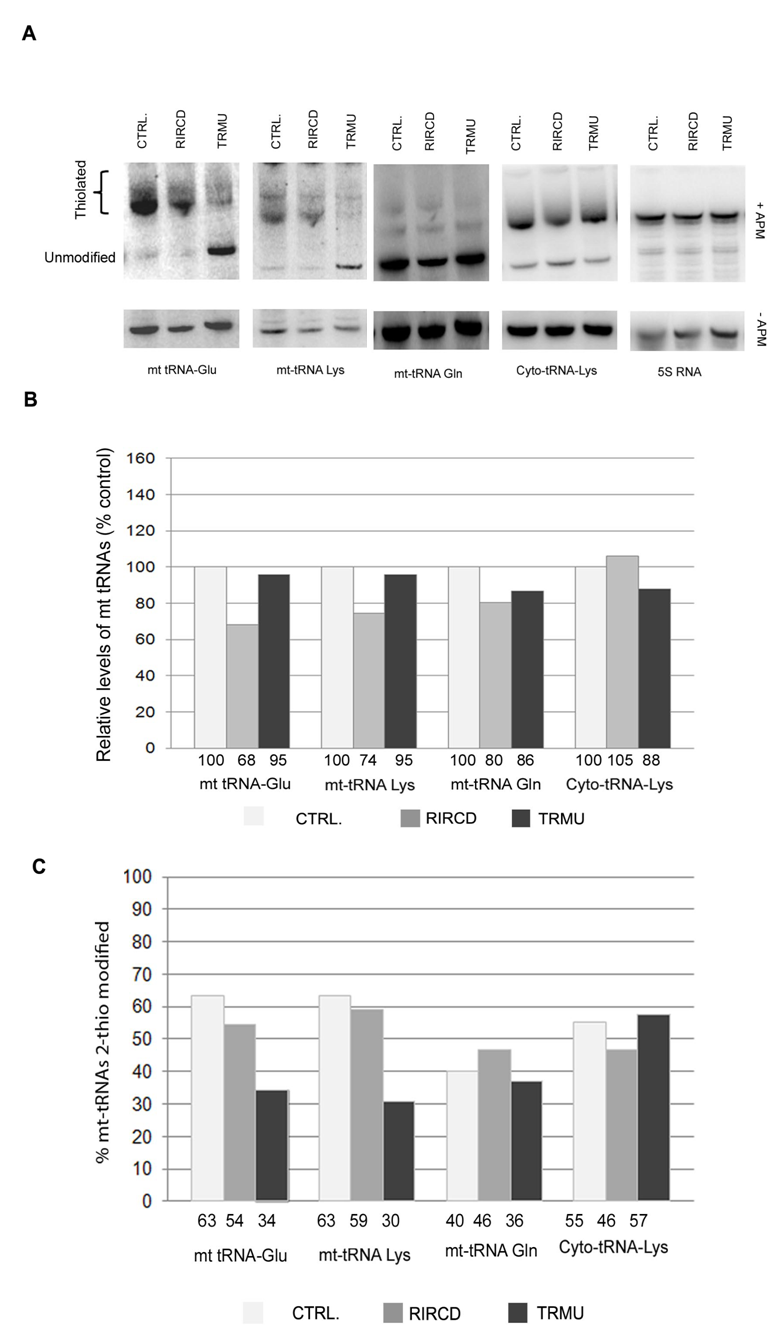


APM: (N-) Acroylamino-phenyl-mercuric chloride). RIRCD: reversible infantile respiratory chain deficiency. TRMU: patient cells carrying the TRMU mutation. CTRL: control.

**A.** Northern blotting with adding APM to the gels to separate thiolated and unthiolated tRNA species was performed and probed for mt-tRNA^Glu^, mt-tRNA^Lys^, mt-trNA^Gln^, cytoplasmic tRNA^Lys^ and 5S rRNA in immortalized human fibroblasts of patients with RIRCD, TRMU deficiency and control cell lines. Results derive from the same experiment, all representative blots were used for all tRNA probes following each other.

**B.** Quantification of the Northern blots shows relative steady-state levels of the tRNAs and

**C.** the % of thiolated tRNA species compared to the whole amount of each tRNAs. For each sample the signal corresponding to the amount of tRNA was normalized to the signal corresponding to the amount of 5S RNA. The total levels of each of the four thio-modified tRNAs in the control cells were set arbitrarily to 100%. The values in the histogram are averages of two measurements, one corresponding to the signal from the gel without APM, the other to the total signal (thiolated plus unmodified) from the gel containing APM. The quantification of the modification is presented at the bottom panel and is expressed as a percentage of the thiolated signal from the thiolated + nonthiolated signals.

**Supplementary Figure 2**

Ablation of TRMU decreased 2-thiouridylation and steady state level of mt-tRNA^Glu^ in RIRCD patient fibroblasts.


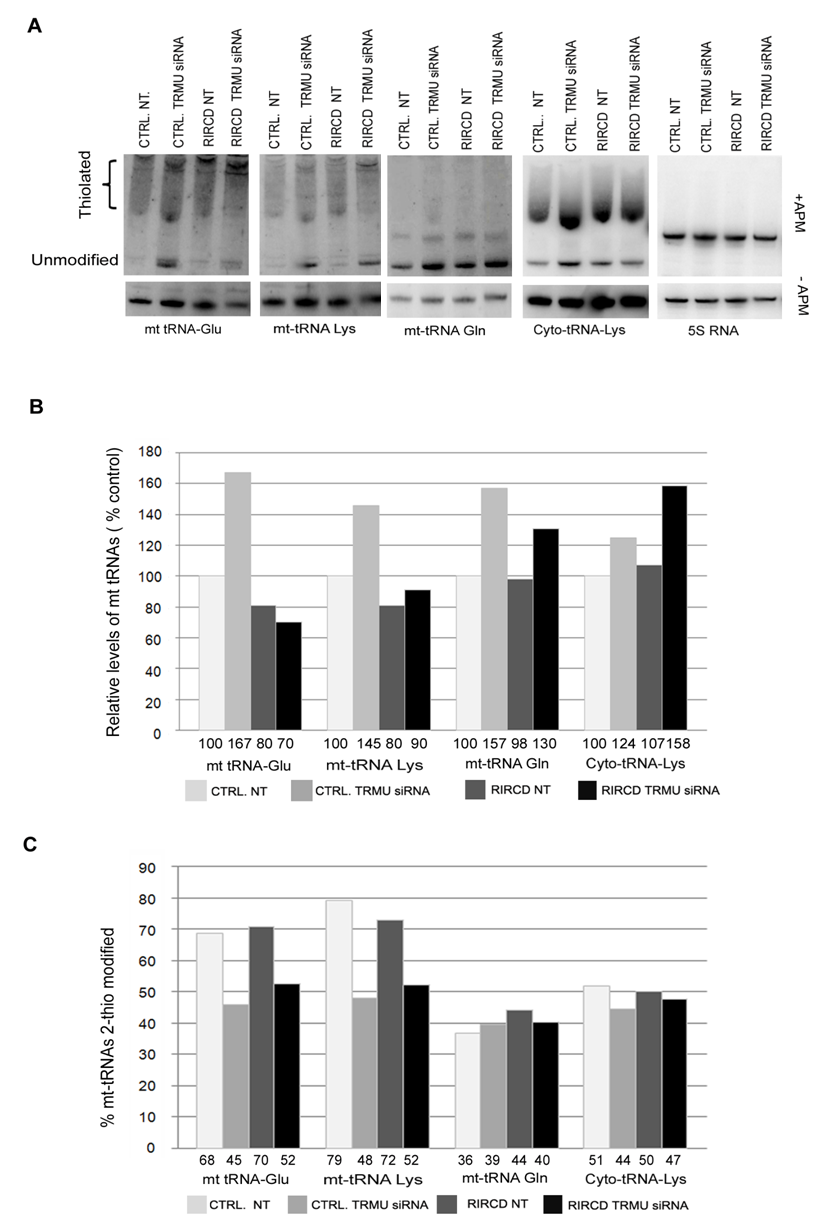


**A.** Northern blotting with/without APM was performed in RIRCD and control fibroblasts after down-regulation of TRMU by siRNA or treatment by non-targeting siRNA (NT). Results derive from the same experiment; blots were used for all tRNA probes subsequently each other. Representative northern blots were quantified as described in Figure 1.

**B.** Relative steady-state levels of the tRNAs.

**C.** We show % of thiolated tRNA species compared to the whole amount of each tRNAs in the studied cell lines.

**Supplementary Figure 3**

Gene expression of *TRMU* in skeletal muscle of controls at different ages (21 days, 3 months, 1 year 6 months, 5 years and adult), muscle biopsy of a child with TRMU deficiency and in RIRCD patient muscle before and after recovery (P1: 1 month, P1: 8 years 9 months).


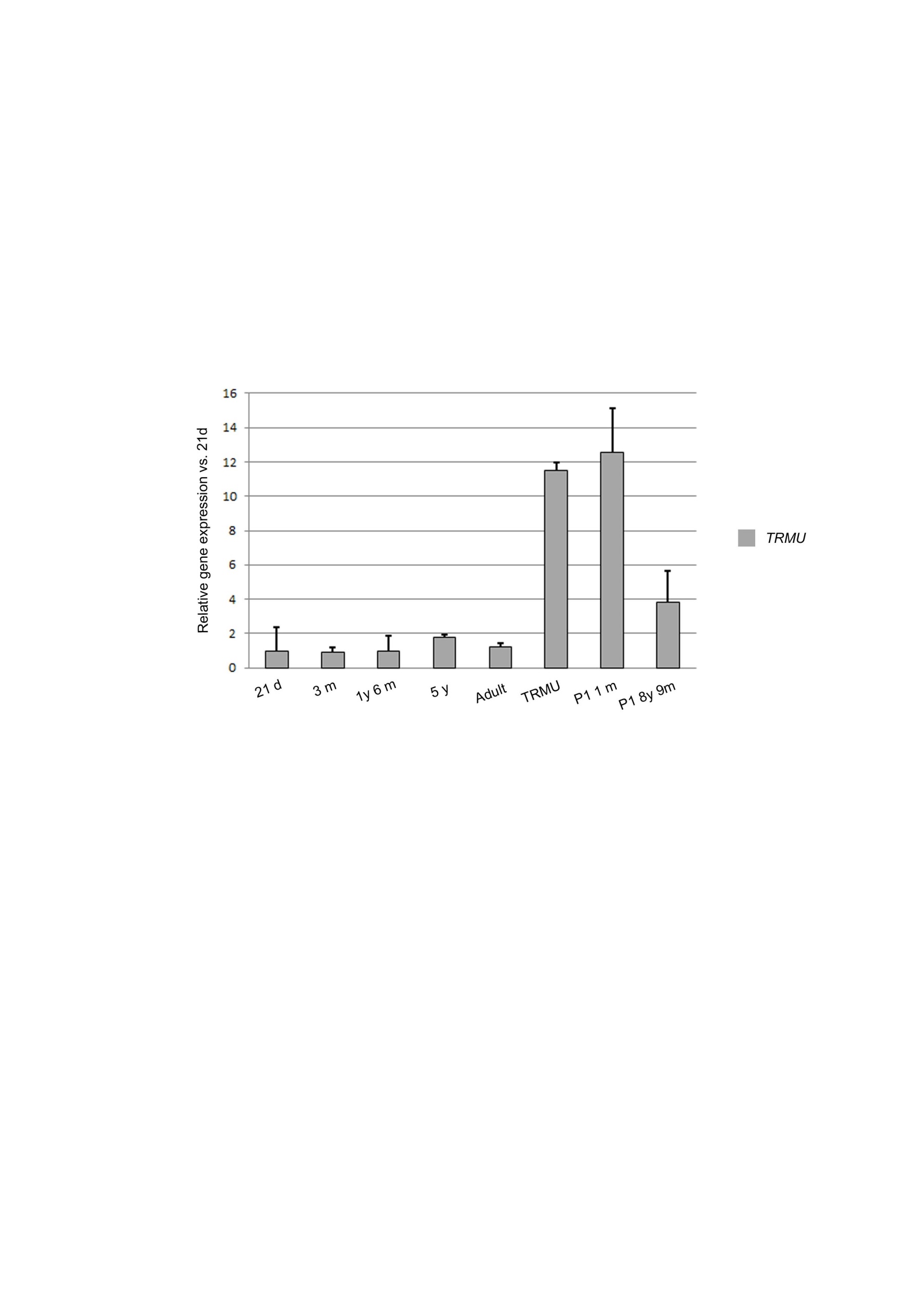


**Supplementary Table 1**

Myoblast and fibroblast cell lines of the following patients were used in the study.

| **Patient cell lines** | **Mutations** |
| --- | --- |
| RIRCD myoblasts (patient 14) and fibroblasts (patient 15)^3^ | Homoplasmic m.14674T>C in mt-tRNA^Glu^ |
| TRMU deficiency^12^ | Compound heterozygous c.711_712insG, p.Gln238AlafsX14 and  c.1081_1082insAGGCTGTGC, p.Arg361 insAla,Val,Arg |
| MTO1 deficiency (unpublished) | Compound heterozygous c.631delG, p.Gly211fs and  c.991G>A, p.Ala331Thr |

**Supplementary Table 2**

Primer sequences used to generate probes for Northern blotting.

|  | **Forward primer** | **Reverse primer** |
| --- | --- | --- |
| mt-tRNA^Glu^ | (mtDNA positions14810–14791)  5*`*-TACTAAACCCACACTCAACAG-3` | (mtDNA positions14810–14791)  5*`*-GGAGGTCGATGAATGAGTGG-3*`* |
| mt-tRNA^Lys^ | (mtDNA positions 8251-8271)  5’-GCCCGTATTTACCCTATAGCA-3’ | (mtDNA positions 8405-8365)  5’-TAATTATGGTGGGCCATACG-3’ |
| mt-tRNA^Gln^ | (mtDNA positions 4307-4329)  5’-AGGAGCTTAAACCCC CTTATTTC-3’ | (mtDNA positions 4456-4434)  5’-GTATAACCAACATTTTCGGGGTA-3’ |
| Cytopl. tRNA^Lys^ | 5’-CCCTCTTATAAGCAAAAAGTAAAG-3’ | 5’-CATTTTATGCGAAGTGCGGTACCC-3’ |
| 5S RNA | 5’-GTCTACGGCCATACCACCCTG-3’ | 5’-AAAGCCTACAGCACCCGGTAT-3’ |

**Supplementary Table 3**

Primer sequences used for RT-PCR studies.

|  | **Forward primer** | **Reverse primer** |
| --- | --- | --- |
| *TRMU* | 5’-TGGTGGAGAAGGACAGCGTCAA-3’ | 5’-TGGCACTCCATCATCTTGTCCC-3` |
| *EARS2* | 5’-AGGTGGATGTGATTGCCAAGCG-3’ | 5’-GTACTTGGTGCCTTCCAGACCT-3’ |
| *MTO1* | 5’-GCATCAGAGGCTTGGAGAAAGC-3’ | 5’-GTGGTGCCATTGATCTGTCCAG-3’ |
| *CTH* | 5’-CTCACTGTCCACCACGTTCAAG-3’ | 5’-CAGTGGCTGCTAAACCTGAAGC-3’ |
| *ACTB* | 5’-GATGCAGAAGGAGATCACTGC-3’ | 5’-ACATCTGCTGGAAGGTGGAC-3’ |
